# Correlations between alexithymia, emotional instability, autism spectrum disorder and eating disorders: analysis of a case

**DOI:** 10.1192/j.eurpsy.2022.1498

**Published:** 2022-09-01

**Authors:** M. Valtueña García, E. Peña Herrero, M. Picado Rossi, C. Pedrosa Duque, P. Valladares Rodriguez

**Affiliations:** 1 Hospital Vital Álvarez-Buylla , Psiquiatria, Asturias, Spain; 2 Hospital de Cabueñes, Psiquiatría, Asturias, Spain; 3 Hospital de Cabueñes, Psicología, Asturias, Spain; 4 Hospital Universitario Central de Asturias, Psicología, Asturias, Spain; 5 Hospital Universitario Central de Asturias, Unidad De Trastornos De La Conducta Alimentaria Huca, Asturias, Spain

**Keywords:** alexithymia, emotional instability, Eating Disorders, Autism Spectrum Disorder

## Abstract

**Introduction:**

Eating disorders and borderline personality disorder can coexist with high frequency in people with alexithymia. At the same time, it has been described that alexithymia can be present in patients suffering from depression, anxiety, obsessive-compulsive disorders, PTSD and eating disorders, among others. In this sense, it has been described that alexithymia could help maintain eating disorder.

**Objectives:**

To review the existing literature on the relationship between alexithymia, emotional instability and a family history of autism spectrum traits with the development of eating disorders. To expose, through the clinical case of a patient with eating disorders, the diagnostic complexity and evolution after the beginning of a comprehensive and multidisciplinary therapeutic plan with different mental health devices.

**Methods:**

To review the personal and family psychopathological aspects and the clinical evolution of a patient with a diagnosis of restrictive subtype anorexia nervosa since its inclusion in a therapeutic program.

**Results:**

This is a longitudinal study through personal biographical reconstruction and family history and subsequent follow-up of a clinical case based on the implementation of an individualized therapeutic program and the results obtained.

**Conclusions:**

Currently there is evidence in the literature that finds a high correlation between alexithymia and eating disorders. However, these findings are believed to be influenced by other comorbid symptoms such as depression or anxiety. Furthermore, the diagnosis of ASD in people with AN is a complex process that requires a thorough clinical evaluation over time. Detailed studies are needed to determine the importance of these factors in the development of an eating disorder.

**Disclosure:**

No significant relationships.

